# Assessment of the association between coronary artery blockage and periodontal health status in patients undergoing coronary angiography: a cross-sectional study

**DOI:** 10.4317/medoral.26946

**Published:** 2025-01-26

**Authors:** Ankita Khandelwal, Raghavendra Vamsi Anegundi, Pradeep Kumar Yadalam, Santhosh B Shenoy, Kodangala Subramanyam, Carlos M Ardila

**Affiliations:** 1 Department of Periodontics, NITTE (Deemed to be University), AB Shetty Memorial Institute of Dental Sciences, Derlakatte, Mangalore, Karnataka, India; 2 Department of Periodontics, Saveetha Dental College, SIMATS, Saveetha University, Chennai, Tamil Nadu India; 3 Department of Cardiology, Srinivas Institute of Medical Sciences and Research Centre, Mukka - Surathkal, India; 4 Basic Sciences Department. Faculty of Dentistry Universidad de Antioquia, UdeA, Medellín, Colombia

## Abstract

**Background:**

Growing evidence suggests a potential link between periodontal disease and the development of atherosclerosis, positioning periodontal disease as a possible risk factor for cardiovascular diseases (CVD). This study aimed to evaluate periodontal status in patients with coronary artery disease (CAD) by measuring the Periodontal Inflamed Surface Area (PISA) score in individuals undergoing coronary angiography.

**Material and Methods:**

In this cross-sectional study, 300 patients scheduled for coronary angiography at K.S. Hegde Medical Hospital, Mangalore, India, were recruited. Comprehensive medical and dental histories were obtained prior to the procedure. The PISA score was calculated using standardized periodontal measurements. Patients were categorized into three groups based on angiographic findings: no coronary artery blockage, blockage in one or more vessels with <50% stenosis, and blockage in one or more vessels with ≥50% stenosis. Additional subgroup analyses were conducted for single-, double-, and triple-vessel disease. A *p-value* of <0.05 was considered statistically significant.

**Results:**

A significant increase in PISA scores was observed in patients with ≥50% coronary artery stenosis compared to those with <50% stenosis and no stenosis. However, the extent of vessel obstruction appeared independent of the degree of periodontal destruction.

**Conclusions:**

This study suggests that periodontal disease may act as a pro-atherogenic factor in the context of CAD, potentially contributing to the progression of atherosclerosis rather than being a direct causative agent. These findings underscore the importance of considering oral health in cardiovascular risk assessment and management for patients with coronary artery disease.

** Key words:**Coronary artery disease, periodontal disease, inflammatory burden, PISA score, risk factor.

## Introduction

Periodontitis is a biofilm-induced, host-mediated immuno-inflammatory condition. Although microbes are closely associated with the disease, the host's immune response plays a central role in the destruction of both hard and soft tissues (1). Evidence suggests that periodontal disease may influence chronic systemic conditions such as diabetes mellitus, coronary heart disease (CHD), stroke, and adverse obstetric outcomes (1,2). This association is often attributed to the systemic inflammatory response triggered by periodontal infections. Cardiovascular diseases (CVDs) are the second leading cause of mortality globally, with atherosclerosis being the primary underlying pathology (3). Atherosclerosis is characterized by chronic, low-grade inflammation that accelerates the progression of existing atheromatous plaques (4).

Offenbacher's model proposes that periodontal disease exacerbates oxidative stress through the production of reactive oxygen species, which oxidize low-density lipoproteins (LDL) (5). These oxidized LDL particles, along with arachidonic acid metabolites, circulate systemically and contribute to atheroma development by promoting foam cell formation, increasing monocyte recruitment, and enhancing the uptake of oxidized LDL. This cascade culminates in plaque formation, which may rupture, leading to thromboembolic events (6).

Inflammatory mediators such as interleukin-1 (IL-1), interleukin-6 (IL-6), tumor necrosis factor-α (TNF-α), C-reactive protein (CRP), and matrix metalloproteinases (MMPs) are released in response to periodontal pathogens. These molecules can enter the systemic circulation, triggering endothelial cells to produce additional inflammatory markers, including monocyte chemoattractant protein-1 (MCP-1), macrophage colony-stimulating factor (M-CSF), vascular cell adhesion molecules, and E-selectin, all of which contribute to atheroma progression (7). Moreover, periodontal pathogens such as *Porphyromonas *gingivalis* (Pg)* and *Aggregatibacter actinomycetemcomitans* (Aa) have been shown to exacerbate atherosclerotic changes in animal models and have been isolated from atheromatous plaques. These bacteria can invade and persist in aortic endothelial cells, promoting platelet adhesion and increasing the risk of thrombus formation (8).

However, conflicting studies suggest that the observed associations may be attributed to shared risk factors between periodontal disease and CVD, rather than a direct causal relationship (9). Furthermore, the varying definitions of periodontal disease and coronary artery disease across studies complicate the interpretation of results, leaving the relationship inconclusive (10).

Few studies have investigated the relationship between angiographically defined coronary artery disease (CAD) and periodontal disease. Those that have typically rely on clinical parameters such as probing pocket depth (PPD) and clinical attachment level (CAL), which may not fully capture the systemic inflammatory burden imposed by periodontitis (11,12).

Therefore, this study aimed to explore the correlation between the severity of coronary artery blockage and the Periodontal Inflamed Surface Area (PISA) (11) score in patients undergoing coronary angiography.

## Material and Methods

- Study Population 

The study was conducted between January 2016 and June 2017 and included 300 patients undergoing coronary angiography at the Cardiology Department of K.S. Hegde Medical Hospital, Deralakatte, Mangalore. Patients were selected based on predefined inclusion and exclusion criteria.

The sample size was calculated using the formula N = 2Sp²[Z1−α/2 + Z1−β/2]²/μd². We used a significance level of α = 0.05, a statistical power of β = 0.80, a pooled variance of Sp² = [SD1² + SD2²]/2, and a clinically meaningful difference of μd = 100 (10). These values resulted in a calculated sample size of 280; however, we enrolled 300 patients to account for potential attrition.

Informed consent was obtained from all participants, and the study protocol was approved by the Institutional Human Ethics Committee of the Memorial Institute of Dental Sciences, Nitte University, India (ABSM/EC/85/15).

A detailed case history, including demographic data (age, sex, and gender), medical background, and smoking status, was recorded before coronary angiography. Body Mass Index (BMI) was calculated by measuring the patient's height and weight using a standardized stadiometer and calibrated weighing scale, with BMI expressed as weight in kilograms divided by height in meters squared (kg/m²). Smoking history was obtained through self-reporting and categorized as current smoker, former smoker, or never smoked. Blood pressure was measured using a calibrated sphygmomanometer after the patient had been seated for 5 minutes, with three readings taken at 1-2-minute intervals, and the average of the last two readings recorded. Fasting blood samples were collected to assess cholesterol levels, including total cholesterol, LDL, HDL, and triglycerides, using standard biochemical assays. Diabetes status was documented based on a prior medical diagnosis and fasting blood glucose levels ≥126 mg/dL, or HbA1c ≥6.5%, according to ADA guidelines (13).

- Selection Criteria

Patients aged 45 to 65 years with a minimum of 20 natural teeth were included in the study. Exclusion criteria encompassed patients with malignancies, tumors, or other cardiac conditions such as congenital heart disease, valvular diseases, and those with prosthetic heart valves.

- Screening Examination 

Before undergoing coronary angiography, all participants underwent a comprehensive dental and periodontal examination conducted by a single examiner to ensure consistency across assessments. Key periodontal parameters, including probing pocket depth (PD), clinical attachment loss (CAL), and bleeding on probing (BOP), were recorded at six sites per tooth (mesial, mid, and distal points on both the buccal and palatal surfaces). Measurements were taken using a William's periodontal probe. Based on these parameters, the Periodontal Inflamed Surface Area (PISA) (11) score was calculated using a pre-designed spreadsheet. This score quantifies the extent of inflamed periodontal tissue by estimating the surface area of the bleeding pocket epithelium in square millimeters. PD was defined as the distance between the gingival margin and the base of the pocket, while CAL was measured as the distance from the cement-enamel junction to the base of the gingival sulcus or pocket.

- Coronary Angiography 

All coronary angiograms were performed by a single cardiologist. The procedure involved inserting a catheter through either the radial or femoral approach and guiding it to the coronary artery openings under fluoroscopic guidance. A contrast medium containing iodine was injected to visualize the coronary arteries and assess the extent and severity of blockages. Angiographic images were recorded digitally for subsequent analysis.

Patients were classified into three groups based on the degree of coronary artery blockage observed in the angiogram reports.

Group A: No coronary artery blockage.

Group B: One or more vessels with less than 50% coronary artery blockage (non-critical group).

Group C: One or more vessels with 50% or greater coronary artery blockage (critical group).

Patients in Groups A and B served as internal controls. Additionally, participants were categorized based on the number of affected vessels into subgroups: no vessel obstruction, single vessel disease, double vessel disease, or triple vessel disease.

- Statistical Analysis 

Data were analyzed using IBM SPSS Statistics, Version 22 (Armonk, NY: IBM Corp). Descriptive statistics were used to summarize the baseline characteristics of the participants, with continuous variables reported as means and standard deviations, and categorical variables as frequencies and percentages.

To assess the normality of data distribution, the Kolmogorov-Smirnov test was employed, revealing a non-normal distribution (*p*<0.05). Consequently, non-parametric tests were utilized in this study. Spearman's correlation coefficients were calculated to assess associations between periodontal parameters and systemic health outcomes. For group comparisons, Mann-Whitney U tests were used for continuous variables, and Kruskal-Wallis tests were applied for multiple group comparisons. The Kruskal-Wallis test evaluates whether there is a statistically significant difference between the distributions of the groups. A significant result (*p*<0.05) indicates that at least one group differs from the others. Post hoc pairwise comparisons were subsequently performed using the Mann-Whitney U test to identify specific group differences, with adjustments made for multiple comparisons (Bonferroni correction) to control for type I error.

Moreover, multivariate regression analysis was performed to adjust for potential confounding factors such as age, gender, BMI, and smoking status. A significance level of *p*<0.05 was applied, and 95% confidence intervals were reported where applicable.

## Results

A total of 45 patients had no affected vessels, 112 had one affected vessel, 90 had two affected vessels, and 53 had three affected vessels. The majority of the study population consisted of male patients (Table 1), with ages ranging from 45 to 65 years (mean age: 55.6 ± 5 years). Most patients did not report a history of smoking. Body Mass Index (BMI) was observed, with most patients falling into the normal BMI category. Vessel obstruction ≥50% was more frequently observed in males, as well as in non-diabetic, non-hypertensive individuals, and those with no history of hypercholesterolemia.

The mean PISA scores for the groups with no vessel obstruction, one vessel obstruction, two vessel obstruction, and three vessel obstruction were 211.21, 754.24, 697.86, and 724.01, respectively. According to the Mann-Whitney U test, PISA scores were significantly higher in patients with single, double, and triple vessel disease compared to those without vessel obstruction (*p*< 0.05). However, no statistically significant differences in PISA scores were found when comparing single, double, and triple vessel stenosis to each other.

When the overall PISA scores were compared across groups with no blockage, <50% blockage, and ≥50% blockage, the scores were 211.21, 424.73, and 893.51, respectively (Table 2). The Kruskal-Wallis test revealed a significant difference in the mean PISA scores across the groups (χ² = 158.94, *p*<0.001). The Mann-Whitney U test revealed that PISA scores were significantly higher in groups with stenosis (*p*<0.05). Additionally, patients with ≥50% blockage had greater periodontal destruction compared to those with <50% blockage.

Spearman’s correlation test demonstrated a significant correlation between PISA scores and both the percentage of coronary artery blockage and the number of vessels blocked (Table 3). This finding indicated that the extent and severity of coronary artery blockage were significantly associated with PISA scores.

In multivariable logistic regression models (Table 4), sex, diabetes, cholesterol levels, hypertension, and smoking history were not significantly associated with the extent of coronary artery disease after adjusting for age. However, PISA scores were significantly associated with higher odds of having ≥50% vessel obstruction.

## Discussion

This study, conducted on a relatively large population (N=300) undergoing coronary angiography, demonstrates a significant association between coronary artery stenosis and periodontal inflammatory burden, as assessed PISA score (11) (Table 2). The relationship between coronary artery blockage and periodontal inflammation remained significant even after adjusting for potential confounders, such as age, sex, smoking status, BMI, and medical history, including diabetes, cholesterol levels, and hypertension, through logistic regression analysis. Notably, while existing literature has established individual links between periodontal disease and systemic conditions, a clear understanding of how the severity of periodontal inflammation correlates with specific metrics of coronary artery disease remains underexplored (12). By addressing this knowledge gap, the present study provides valuable insights into the systemic inflammatory interactions between periodontitis and coronary artery disease, potentially elucidating pathways that could inform clinical practice and future research.

The strength of this association was further highlighted by Spearman’s correlation coefficient, which demonstrated a significant positive correlation between PISA scores and both the number of vessels blocked and the percentage of coronary artery stenosis (Table 3). These findings are consistent with previous studies, such as the one by Ketabi *et al*., which showed that clinical periodontal parameters, such as pocket depth (PD) and clinical attachment loss (CAL), were significantly higher in patients with ≥50% coronary artery blockage compared to those with <50% blockage or no blockage (14). Similarly, Amabile *et al*. demonstrated a positive correlation between the severity of coronary artery disease (CAD) and periodontal probing depth, along with elevated systemic biomarkers like high-sensitivity C-reactive protein (hs-CRP) and fibrinogen, suggesting a dose-response relationship between the two conditions (15).

The observed higher PISA scores in patients with severe coronary artery blockage likely reflect the role of periodontal infection in exacerbating systemic inflammation, contributing to the progression of atherosclerosis. Periodontal pathogens may enter the bloodstream and release pro-inflammatory molecules, such as prostaglandins, IL-1, and TNF-α, which could further promote atherogenesis and vascular damage (16,17).

Interestingly, while PISA scores were significantly elevated in patients with coronary stenosis compared to those without, there was no significant difference in PISA scores when comparing patients with single, double, and triple vessel disease. This suggests that while periodontal inflammation is associated with the presence and severity of coronary blockage, it may not directly influence the number of affected vessels. Notably, patients with ≥50% blockage exhibited significantly higher PISA scores compared to those with <50% blockage, indicating that periodontal destruction is linked to more severe coronary artery obstruction. This supports findings from previous studies, which have shown worse outcomes in CAD patients with more than 50% vessel blockage.

The validity of PISA as a tool to measure periodontal infection and its systemic impact has been supported in various studies. For instance, Muthukumar *et al*. demonstrated that PISA, along with the BANA test, could indicate the presence of anaerobic bacteria in periodontitis patients (18). Other studies have also used PISA to quantify the systemic inflammatory burden in conditions such as diabetes. Researchers like Nesse *et al*. (19) and Susanto *et al*. (20) have confirmed the utility of PISA in assessing the inflammatory burden caused by periodontal disease, which may also reflect its impact on systemic health, including cardiovascular disease.

Periodontitis and CAD share common risk factors, including smoking, age, stress, and systemic diseases like diabetes and dyslipidemia (21). In this study, multivariate regression models were used to control for these confounders, revealing that higher PISA scores were significantly associated with CAD (Table 4). However, the odds ratio for this association was modest (OR=1.01), suggesting that while periodontal inflammation is a contributing factor, it may not be the primary driver of CAD. This finding aligns with a study by Chrysanthakopoulos *et al*., which also demonstrated an association between periodontal parameters and CAD, although with a higher odds ratio (2.38) (22). The difference in odds ratios could be attributed to the self-reported CAD history in the previous study, whereas the present study exclusively considered angiographically confirmed CAD cases.

An intriguing observation in this study is that while periodontal destruction was linked to the severity of coronary blockage, it did not influence the number of vessels obstructed. This implies that periodontal infection may exacerbate existing atherosclerosis rather than act as a direct etiological factor in the initiation of atherosclerosis. The higher PISA scores observed in patients with ≥50% blockage suggest that the inflammatory burden from periodontal disease may have a pro-atherogenic effect, contributing to increased coronary obstruction.

The findings of this study suggest that periodontal disease, as reflected by the PISA score, may serve as an important indicator of systemic inflammatory burden in patients with CAD. Given the significant association between periodontal inflammation and coronary artery blockage, early diagnosis and management of periodontal disease could potentially reduce the progression of atherosclerosis and improve cardiovascular outcomes. Periodontal interventions may be particularly beneficial in high-risk individuals, such as those with ≥50% coronary artery stenosis, to mitigate systemic inflammation and its pro-atherogenic effects.

This study's strengths include a large sample size and the use of the PISA score, a comprehensive metric for assessing periodontal disease. Since PISA quantifies the inflamed periodontal tissue, it likely provides an estimate of the systemic inflammatory burden resulting from periodontal disease. Furthermore, the full-mouth PISA score minimizes potential biases related to disease activity estimation.

A key limitation of the present study is the reliance on internal controls (Groups A and B), which consisted of patients with no or non-critical coronary artery blockage. Although this approach allowed us to explore the relationship between periodontal inflammation and varying degrees of CAD, the lack of an external control group composed of individuals without any clinical or angiographic evidence of CAD limits the strength of our conclusions. Including such a non-CAD control group in future studies would strengthen the comparative analysis and offer more definitive insights into the potential link between periodontal health and cardiovascular risk. Additionally, the cross-sectional design of this study restricts our ability to establish a causal relationship between periodontitis and CAD. Longitudinal studies are needed to determine whether periodontal disease contributes directly to the progression of CAD.

Moreover, the inclusion of systemic biomarkers like hs-CRP, IL-6, and fibrinogen, along with detailed assessments of coronary artery lesion characteristics and calcifications, would provide deeper insights into the mechanisms linking periodontal disease and atherosclerosis. Future research incorporating these factors could enhance our understanding of how periodontal parameters influence cardiovascular health and improve the predictive value of periodontal health in cardiovascular risk assessment.

## Conclusions

In conclusion, this study establishes a compelling link between periodontal inflammatory burden, as quantified by the PISA score, and the severity of coronary artery disease, highlighting the intricate interplay between oral health and systemic conditions. The significant correlation between increased periodontal inflammation and coronary artery stenosis underscores the importance of integrated healthcare approaches that consider oral health as a vital component of cardiovascular risk assessment and management. These findings reinforce the role of periodontal disease as a potential contributor to systemic inflammation and atherogenesis, while also emphasizing the need for further research to elucidate the underlying mechanisms of this relationship. By advancing our understanding of the association between periodontitis and coronary artery disease, this study paves the way for novel preventive and therapeutic strategies aimed at mitigating cardiovascular risk in individuals with periodontal disease, ultimately improving patient outcomes across both domains of health.

## Figures and Tables

**Table 1:** Distribution of patients according to independent variables and severity of vessel obstruction.

Variable	Categories	Severity			Total	Chi square test	
		No obstruction	<50% obstruction	≥50% obstruction		Chi square vale	*p-value*
Sex	F	15	16	68	99	0.34(2)	0.84 (NS)
	M	30	38	133	201		
Age	<60	26	38	147	211	4.16	0.13 (NS)
	>60	19	16	54	89		
Smoking history	Never	30	37	143	210	-	0.78 (NS)^ #^
	Former	2	4	8	14		
	Current	13	13	50	76		
BMI	Underweight	1	1	1	3	-	0.03*^#^
	Normal	34	29	104	167		
	Overweight	10	23	93	126		
	Obese	0	1	3	4		
Normal Blood Pressure	No	35	32	144	211	4.53	0.10 (NS)
	Yes	10	22	57	89		
Diabetic	No	34	47	152	233	3.33	0.19 (NS)
	Yes	11	7	49	67		
Cholesterol	No	35	43	149	227	0.83	0.66 (NS)
	Yes	10	11	52	73		

# Fisher's exact test; *p<0.05 statistically significant; *p*>0.05 non-significant, NS.

**Table 2:** Comparison of PISA score between no blockage, <50% blockage and ≥50% blockage.

% of blockage	N	Mean PISA score	Kruskal-Wallis Test		Mann Whitney U test (*p-value*)		
			Chi square value	*p-value*	0 vs <50%	0 vs ≥50%	<50% vs ≥50%
0	45	211.21 (147.28)	158.9	<0.001*	<0.001*	<0.001*	<0.001*
<50%	90	424.73 (216.48)					
≥50%	165	893.51 (445.46)					

**Table 3:** Correlation between PISA score and percentage of coronary artery blockage.

Parameter		Total cholesterol	Triglyceride	LDL	HDL	PISA score	Percentage
Triglyceride	Correlation Coefficient	0.53	1.00	-	-	-	-
	*p-value*	<0.001*	-	-	-	-	-
LDL	Correlation Coefficient	0.54	0.53	1.00	-	-	-
	*p-value*	<0.001*	<0.001*	-	-	-	-
HDL	Correlation Coefficient	-0.33	-0.41	-0.37	1.00	-	-
	*p-value*	<0.001*	<0.001*	<0.001*	-	-	-
PISA score	Correlation Coefficient	0.06	0.05	0.09	-0.05	1.00	-
	*p-value*	0.34 (NS)	0.42 (NS)	0.14 (NS)	0.42 (NS)	-	-
Percentage	Correlation Coefficient	0.07	0.01	0.06	-0.11	0.76	1.00
	*p-value*	0.25 (NS)	0.86 (NS)	0.28 (NS)	0.06 (NS)	<0.001*	-
No. of vessels	Correlation Coefficient	0.03	0.04	0.09	-0.06	0.27	0.22
	*p-value*	0.61 (NS)	0.52 (NS)	0.12 (NS)	0.32 (NS)	<0.001*	<0.001*

*p<0.05 statistically significant; *p*>0.05 non-significant, NS.

**Table 4:** Multivariate binary logistic regression models of the association between severity of coronary artery blockage and other independent variables.

Parameters	Beta	Standard error	Wald	p-value*	Odds Ratio	95% confidence interval for Odds Ratio	
						Lower	Upper
PISA score	0.01	0.001	41.08	<0.001*	1.01	1.01	1.01
BMI	0.25	0.08	9.23	0.002*	1.29	1.09	1.51
Sex (Female)	0.42	0.53	0.64	0.43 (NS)	1.52	0.54	4.28
Diabetic (present)	-0.27	0.54	0.24	0.63 (NS)	0.77	0.27	2.22
Cholesterol (present)	-0.13	0.58	0.05	0.82 (NS)	0.88	0.28	2.72
Hypertension (present)	1.01	0.56	3.26	0.07 (NS)	2.75	0.92	8.26
Never smokers	-	-	1.43	0.49 (NS)	-	-	-
Former smokers	-0.80	1.09	0.54	0.46 (NS)	0.45	0.05	3.81
Current smokers	-0.52	0.50	1.12	0.29 (NS)	0.59	0.23	1.56
Constant	-2.72	2.94	0.86	0.36 (NS)	0.07	-	-

Model chi- square (10) =119.35 (*p*<0.001); -2 Log likelihood- 134.28; Cox & Snell R^2^ = 0.33; Nagelkerke R^2^ = 0.58.

## References

[1] Abdulkareem AA, Al-Taweel FB, Al-Sharqi AJ, Gul SS, Sha A, Chapple IL (2023). Current concepts in the pathogenesis of periodontitis: from symbiosis to dysbiosis. J Oral Microbiol.

[2] Kim J, Amar S (2006). Periodontal disease and systemic conditions: a bidirectional relationship. Odontology.

[3] Zhu M, Jin W, He W, Zhang L (2024). The incidence, mortality and disease burden of cardiovascular diseases in China: a comparative study with the United States and Japan based on the GBD 2019 time trend analysis. Front Cardiovasc Med.

[4] Amato M, Lupi SM, Polizzi A, Santonocito S, Viglianisi G, Cicciù M (2023). New Trends in the Impact of Periodontal Treatment on Early Cardiovascular Diseases Outcomes: Insights and Future Perspectives. Rev Cardiovasc Med.

[5] Offenbacher S, Madianos PN, Champagne CM, Southerland JH, Paquette DW, Williams RC (1999). Periodontitis-atherosclerosis syndrome: an expanded model of pathogenesis. J Periodontal Res.

[6] Jebari-Benslaiman S, Galicia-García U, Larrea-Sebal A, Olaetxea JR, Alloza I, Vandenbroeck K (2022). Pathophysiology of Atherosclerosis. Int J Mol Sci.

[7] Cecoro G, Annunziata M, Iuorio MT, Nastri L, Guida L (2020). Periodontitis, Low-Grade Inflammation and Systemic Health: A Scoping Review. Medicina (Kaunas).

[8] Ruan Q, Guan P, Qi W, Li J, Xi M, Xiao L (2023). Porphyromonas gingivalis regulates atherosclerosis through an immune pathway. Front Immunol.

[9] Sessa R, Pietro MD, Filardo S, Turriziani O (2014). Infectious burden and atherosclerosis: A clinical issue. World J Clin Cases.

[10] Beck JD, Philips K, Moss K, Sen S, Morelli T, Preisser J (2020). Periodontal disease classifications and incident coronary heart disease in the Atherosclerosis Risk in Communities study. J Periodontol.

[11] Nesse W, Abbas F, van der Ploeg I, Spijkervet FK, Dijkstra PU, Vissink A (2008). Periodontal inflamed surface area: quantifying inflammatory burden. J Clin Periodontol.

[12] Febbraio M, Roy CB, Levin L (2022). Is There a Causal Link Between Periodontitis and Cardiovascular Disease? A Concise Review of Recent Findings. Int Dent J.

[13] Weir MR (2024). Cardiovascular risk reduction in type 2 diabetes: What the non-specialist needs to know about current guidelines. Diabetes Obes Metab.

[14] Ketabi M, Meybodi FR, Asgari MR (2016). The association between periodontal disease parameters and severity of atherosclerosis. Dent Res J (Isfahan).

[15] Amabile N, Susini G, Pettenati-Soubayroux I, Bonello L, Gil JM, Arques S (2008). Severity of periodontal disease correlates to inflammatory systemic status and independently predicts the presence and angiographic extent of stable coronary artery disease. J Intern Med.

[16] Choi H, Dey AK, Priyamvara A, Aksentijevich M, Bandyopadhyay D, Dey D (2021). Role of Periodontal Infection, Inflammation and Immunity in Atherosclerosis. Curr Probl Cardiol.

[17] Sanz M, Marco Del Castillo A, Jepsen S, Gonzalez-Juanatey JR, D'Aiuto F, Bouchard P (2020). Periodontitis and cardiovascular diseases: Consensus report. J Clin Periodontol.

[18] Muthukumar S, Anand MV, Madhankumar S (2014). Relationship between gingival bleeding and anaerobic periodontal infection assessed by BANA (N-Benzoyl-DL-Arginine-β-Napthylamide) assay. J Pharm Bioallied Sci.

[19] Nesse W, Linde A, Abbas F, Spijkervet FK, Dijkstra PU, de Brabander EC (2009). Dose-response relationship between periodontal inflamed surface area and HbA1c in type 2 diabetics. J Clin Periodontol.

[20] Susanto H, Nesse W, Dijkstra PU, Hoedemaker E, van Reenen YH, Agustina D (2012). Periodontal inflamed surface area and C-reactive protein as predictors of HbA1c: a study in Indonesia. Clin Oral Investig.

[21] Herrera D, Sanz M, Shapira L, Brotons C, Chapple I, Frese T (2023). Association between periodontal diseases and cardiovascular diseases, diabetes and respiratory diseases: Consensus report of the Joint Workshop by the European Federation of Periodontology (EFP) and the European arm of the World Organization of Family Doctors (WONCA Europe). J Clin Periodontol.

[22] Chrysanthakopoulos NA, Chrysanthakopoulos PA (2013). Association of periodontal disease with self-reported systemic disorders in Greece. Oral Health Prev Dent.

